# Spatiotemporal modeling of ecological and sociological predictors of West Nile virus in Suffolk County, NY, mosquitoes

**DOI:** 10.1002/ecs2.1854

**Published:** 2017

**Authors:** Mark H. Myer, Scott R. Campbell, John M. Johnston

**Affiliations:** 1 Oak Ridge Institute for Science and Education, Office of Research and Development, National Exposure Research Laboratory, U.S. Environmental Protection Agency, 960 College Station Road, Athens, Georgia 30605 USA; 2 Arthropod-Borne Disease Laboratory, Suffolk County Department of Health Services, Yaphank, New York 11980 USA; 3 Office of Research and Development, National Exposure Research Laboratory, U.S. Environmental Protection Agency, 960 College Station Road, Athens, Georgia 30605 USA

**Keywords:** Bayesian, *Culex pipiens*, disease ecology, integrated nested Laplace approximation (INLA), Long Island, septic systems, spatial modeling, spatiotemporal modeling, stochastic partial differential equation (SPDE), Suffolk, West Nile

## Abstract

Suffolk County, New York, is a locus for West Nile virus (WNV) infection in the American northeast that includes the majority of Long Island to the east of New York City. The county has a system of light and gravid traps used for mosquito collection and disease monitoring. In order to identify predictors of WNV incidence in mosquitoes and predict future occurrence of WNV, we have developed a spatiotemporal Bayesian model, beginning with over 40 ecological, meteorological, and built-environment covariates. A mixed-effects model including spatially and temporally correlated errors was fit to WNV surveillance data from 2008 to 2014 using the R package “R-INLA,” which allows for Bayesian modeling using the stochastic partial differential equation (SPDE) approach. The integrated nested Laplace approximation (INLA) SPDE allows for simultaneous fitting of a temporal parameter and a spatial covariance, while incorporating a variety of likelihood functions and running in R statistical software on a home computer. We found that land cover classified as open water and woody wetlands had a negative association with WNV incidence in mosquitoes, and the count of septic systems was associated with an increase in WNV. Mean temperature at two-week lag was associated with a strong positive impact, while mean precipitation at no lag and one-week lag was associated with positive and negative impacts on WNV, respectively. Incorporation of spatiotemporal factors resulted in a marked increase in model goodness-of-fit. The predictive power of the model was evaluated on 2015 surveillance results, where the best model achieved a sensitivity of 80.9% and a specificity of 77.0%. The spatial covariate was mapped across the county, identifying a gradient of WNV prevalence increasing from east to west. The Bayesian spatiotemporal model improves upon previous approaches, and we recommend the INLA SPDE methodology as an efficient way to develop robust models from surveillance data to develop and enhance monitoring and control programs. Our study confirms previously found associations between weather conditions and WNV and suggests that wetland cover has a mitigating effect on WNV infection in mosquitoes, while high septic system density is associated with an increase in WNV infection.

## Introduction

West Nile virus (WNV) is considered an emerging health threat in the United States, where the first human case occurred in 1999 in New York City (Lanciotti et al. 1999). This pathogen is an arbovirus (family *Flaviviridae*, genus *Flavivirus*), which is vectored by mosquitoes and has become seasonally endemic in the northeastern United States (Hayes et al. 2005), with human cases recurring yearly in the summer and fall (Cruz-Pacheco et al. 2009). From 2008 to 2015, there were 65 reported human WNV infections in Suffolk County, New York, resulting in three deaths (Suffolk County Department of Health Services, *unpublished* data). In the same time span, WNV was detected in 650 birds and two horses. As WNV spreads to affect greater numbers of communities throughout North and Central America, it becomes increasingly important for epidemiologists and public health professionals to understand the geographical, meteorological, and sociological factors that correlate with the presence of the disease. Additionally, the 2015–2016 emergence of the Zika virus in the Western Hemisphere has led to a surge of interest in spatial predictive modeling of mosquito-vectored diseases, especially those models based upon easily obtainable public datasets (Caminade et al. 2016). A robust set of models for predicting WNV prevalence in vectors and reservoir hosts will contribute to identification of potential hotspots before outbreaks occur, allowing preventive action to be taken.

In the northeastern United States, the primary vectors of WNV have been identified as *Culex pipiens*, ornithophilic mosquitoes that transfer the virus from bird reservoirs to humans (Kilpatrick et al. 2005, Turrell et al. 2005). As humans are dead-end hosts of WNV, there is no further contagious spread of the disease once a person is infected (Baum 2008). Because of this, the prevention of WNV focuses on eliminating mosquito vectors and avoiding their bites. *Culex* species are associated with urbanized areas and breed prolifically in organically enriched fresh water (Pratt and Moore 1993). Factors that influence abundance of *Culex* mosquitoes include a positive correlation with impervious cover and urban land use (Diuk-Wasser et al. 2006), and increased abundance when a wet winter and spring are followed by a dry summer (Shaman et al. 2011). In dry summers, birds that serve as reservoir hosts for WNV cluster around eutrophic water sources, exposing them to mosquito carriers. While the presence of wetlands and regularly flooded areas increases the populations of many potential WNV vector mosquitoes (Diuk-Wasser et al. 2006), evidence suggests that populations of *Culex* species responsible for WNV transmission in Long Island have a negative association with healthy, well-functioning wetlands (Carver et al. 2015). *Culex* species avoid laying their eggs in areas with predators (Blaustein et al. 2005, Walton et al. 2009), preferring water that is too polluted to support predators and competitors (Agnew et al. 2000, Chaves et al. 2009). Larval survival of *Culex quinquefasciatus* increased from 2.6% in water bodies containing natural predators to 46% in those that excluded them (Marten et al. 2000), with excessive pollution excluding predatory copepods.

A variety of statistical approaches have been taken to model the spread and prevalence of WNV; however, these all have limitations. In practice, one must choose between employing a temporal or spatial approach, as including both is statistically complex and computationally intensive. In the last decade, a common approach has been linear regressive modeling using variously transformed mosquito count data as the response to estimate the abundance of competent vectors (Diuk-Wasser et al. 2006, Liu et al. 2009, Trawinski and Mackay 2010), although other responses such as percentage of WNV-positive mosquitoes among those trapped (Shaman et al. 2011), absolute number of WNV-positive hosts (Yoo 2014), and odds ratios of human WNV infection (Brown et al. 2008) have been used. Spatial approaches have included mapping data to a grid (Diuk-Wasser et al. 2006, Little et al. 2016), aggregating data by some areal unit such as a county or ZIP code (Young et al. 2013), or the use of cluster analysis such as a Kernel function to detect disease clusters (Vazquez-Prokopec et al. 2010). These approaches attempt to solve the small numbers problem prevalent in disease mapping, in which cases must be aggregated because they are too rare or spatially dispersed to reliably predict disease rates (Pringle 1995). The simplest approach to addressing temporal patterns in WNV is to lag some or all predictor variables, with a lag time chosen according to the ecology of the disease (Shaman et al. 2011, Yoo 2014). Lagging variables does not provide information on the degree of temporal correlation that is present over time, however. For this reason, time series models such as the autoregressive moving average are often used (Trawinski and Mackay 2008). Studies that include both spatial and temporal modeling of WNV have either used separate spatial and temporal analyses which were then combined for inference (Trawinski and Mackay 2010) or employed complex statistical approaches that are not easily applied using commonly used statistical software (Yoo 2014).

Due to regional variation in endemic mosquito species, models of WNV prevalence are typically not generalizable beyond their original geographical scope (Bowden et al. 2011), fostering a need for continual modeling efforts in new areas. Additionally, as climate change slowly impacts the extent of climate zones, and hence the distribution of endemic mosquito species, old WNV models may need to be updated. There is a need for a generally applicable methodology that is computationally efficient while facilitating the evaluation of potentially a large number of spatial and temporal covariates in a combined model.

The integrated nested Laplace approximation (INLA) modeling approach provides the ability to use Bayesian inference with a latent Gaussian model fit to large datasets in a short time while using fewer computing resources than commonly used approaches such as the WinBUGS or JAGS Gibbs samplers, which use the more standard and time-consuming Markov chain Monte Carlo algorithms (Rue et al. 2009). The INLA approach is particularly well suited to spatial and temporal models of disease incidence, because they are usually described using latent Gaussian models with a hierarchical Bayesian framework (Schrodle and Held 2011). Large computational times remain a major drawback of describing spatial data using a Gaussian random field. However, a recent solution has been developed using a stochastic partial differential equation (SPDE) solution to provide explicit links between Gaussian random fields and the much simpler-to-compute Gaussian Markov random fields (Lindgren et al. 2011). Disease ecology models often suffer from the problem of rare events, which can limit spatiotemporal analysis by necessitating clustering or binning responses in space to achieve sufficient resolution. By utilizing Gaussian random fields, SPDE allows the user to map the spatial covariate over any desired spatial resolution, providing a solution for rare events prediction. The SPDE solution allows spatial modeling on a Gaussian random field with computational resources found on a typical personal computer.

In this study, we used a Bayesian INLA SPDE method to fit a spatiotemporal model of WNV infection rates in Suffolk County, Long Island, mosquitoes. By utilizing easily obtained covariates from public data sources along with a county-provided mosquito trapping dataset, we provide a model that can be used by local municipalities to prioritize and target WNV-preventive efforts.

## Methods

### Study area

Suffolk County, New York, USA, covers the eastern portion of Long Island, with a land area of approximately 2362 km^2^. The 2015 United States Census estimated a population density of 635.7 persons per square km, with a total population of 1,501,587 inhabitants. The county is 138 km in length and borders several large bodies of water including the Long Island Sound to the north and the Atlantic Ocean to the south. Major land-cover types include urban/suburban development, which is prevalent in the western half of the county, and wetlands, deciduous forest, and cropland in the more rural east. Between 1981 and 2010, the annualized mean temperature ranged from −0.75°C in January to 23.25°C in July, with 1174.5 mm of average annual precipitation (National Oceanic and Atmospheric Administration 2016). The climate is classified as a transition zone between the humid subtropical and humid continental Köpen-Geiger climate classifications (Peel et al. 2007). As a result of a high water table and hundreds of years of dense human habitation, septic systems are unusually prevalent in Suffolk County. An estimated 74% of housing units are not served by a sewer system, compared to approximately 24% nationally in 1990, the last year sewer data were collected by the U.S. Census (United States Census Bureau 2011). Further, 252,530 homes are served by old cesspool systems not meeting current wastewater standards, representing approximately two-third of unsewered parcels (Suffolk County Department of Environmental Quality 2015). These systems contribute to nitrogen pollution of the aquifer, leading to estuarine and wetland degradation, algal blooms, and drinking water concerns. Septic systems and cesspools are known to provide a predator-free and sheltered habitat for mosquito breeding (Burke et al. 2010).

### Data sources

Mosquito testing data from 2008 to 2015 were provided by the Suffolk County Department of Health Services, Arthropod-Borne Disease Laboratory (Table 1). Trapping was conducted from June through October at a total of 305 locations over the study period, with a mean 67 ± 16.74 (standard deviation [SD]) traps deployed each year. Trap site locations varied by year, guided by WNV surveillance in humans and mosquitoes as each season progressed. Paired CDC light traps and gravid traps (John W. Hock, Gainesville, Florida, USA) were baited with CO_2_ and rabbit chow infusion, respectively, and placed within 5 m of the trap site’s recorded coordinates. Traps were checked weekly. Trapped mosquitoes were sacrificed with dry ice and identified to species as possible. *Culex* mosquito species are extremely morphologically similar, and can be difficult or impossible to differentiate without genetic examination if damage to key characters occurs in trapping or transport (Harrington and Poulson 2008, Rudolf et al. 2013). For this reason, *Culex pipiens, Culex restuans*, and *Culex salinarius* mosquitoes are combined for the purposes of arboviral testing in New York (Bernard and Kramer 2001). For the purposes of this study, only *Culex* mosquito pools were considered, with other mosquitoes such as *Aedes albopictus* and *Aedes vexans* set aside. Mosquito pools were submitted to the New York State Department of Health (Arbovirus Laboratory, Wadsworth Center) to be tested for the presence of WNV using reverse transcription polymerase chain reaction.

Meteorological variables were obtained from the NASA Daymet daily surface weather and climatological summary dataset (Thornton et al. 1997). Daymet has a 1-km spatial resolution and provides daily modeled estimates of precipitation, temperature, radiation, and vapor pressure across North America and Hawaii. Data were downloaded and sorted using the “daymetr” package in R (Hufkens 2014). Geolocations of each trap site were provided, and weather data for the corresponding pixel and day were downloaded individually for each trap site. Variables considered included weekly mean temperature and weekly mean precipitation. The daily weather data were grouped into weekly means by taking the arithmetic mean of the seven days within the numerical week (1 through 52) that each observation fell within. An approximation of weekly average temperature was derived by taking the mean of the weekly maximum and minimum temperatures as reported by Daymet (Depradine and Lovell 2004).

Ecological factors included land cover, Normalized Difference Vegetation Index (NDVI), and soil type. Land cover was obtained from the 2011 National Land Cover Database (Homer et al. 2015), with a spatial resolution of 30 m. All raster data layers were managed and manipulated using ArcGIS 10.3 (Environmental Systems Research Institute, Redlands, California, USA). A zonal statistics function was used to determine the percent cover of each of 16 land-use/land-cover types within a 1-km buffer around each trap site. One kilometer was chosen as the buffer size based on the findings of Trawinski and Mackay (2010), in which land-cover variables gathered with a range of buffer sizes between 200 and 1000 m were tested for correlation to population abundance for several mosquito species. Their results indicated that 1 km was the optimal buffer size for land-cover variables associated with *C. pipiens–restuans* population. The land-cover percentages derived from the zonal function were considered as input variables (Table 2). Normalized Difference Vegetation Index within a 1-km buffer was obtained from NASA Landsat 8 scenes, downloaded as a 30-m raster using Landsat 8 Image Service Add-In for ArcGIS. Soil type data were obtained as a 30-m raster from the Suffolk County Arthropod-Borne Disease Laboratory, sourced from the U.S. Department of Agriculture Soil Survey Geographic (SSURGO) database (USDA NRCS 2016).

As a sociological/anthropogenic variable, we included the estimated number of septic systems within a 1-km buffer of each trap site. In Puerto Rico, poorly maintained and inadequately covered septic tanks were found to contribute to the production of *Culex* mosquitoes, though studies in the area were focused on *Aedes* mosquitoes that transmit dengue fever (Barrera et al. 2008, Burke et al. 2010). Similar associations between septic systems and vector mosquitoes have been found in India (Yadav et al. 1997), Turkey (Cetin et al. 2007), and South Korea (Chae et al. 2014). The number of unsewered parcels in Suffolk County was estimated by superimposing a polygon map of sewered areas and a map of all residential parcels. Parcels that lay along the boundary of the sewering polygon were sorted using the location of the parcel centroid. A focal statistics tool was used to count the residential parcels lying in unsewered areas within a 1-km buffer of each trap site.

### Statistical analysis

Initial predictors were chosen by preparing a correlation matrix of non-meteorological variables (Data S1) to identify variables that were highly correlated, and eliminating those that were correlated with another predictor (correlation coefficient >0.5) by choosing one predictor among the correlated group. This step was taken as an initial precaution against multicollinearity prior to principal component analysis (PCA). Principal component analysis was performed with the remaining predictors and the resulting components were used to eliminate redundant variables. Variables within components were chosen holistically based on the strength of their contribution to the component, availability and spatiotemporal resolution of the variable, and confidence in the accuracy of the variable. For example, two variables, NDVI and Loamy Soil, were grouped in the same principal component with a similar magnitude, possibly because loamy soil supports thick plant cover. Normalized Difference Vegetation Index was chosen rather than Loamy Soil, because remote-sensed NDVI measurements have a better temporal coverage than SSURGO soil surveys. Remaining predictors were normalized by subtracting the mean value and dividing by the SD in order to aid in model convergence and interpretation of coefficients.

**Fig. 1.**
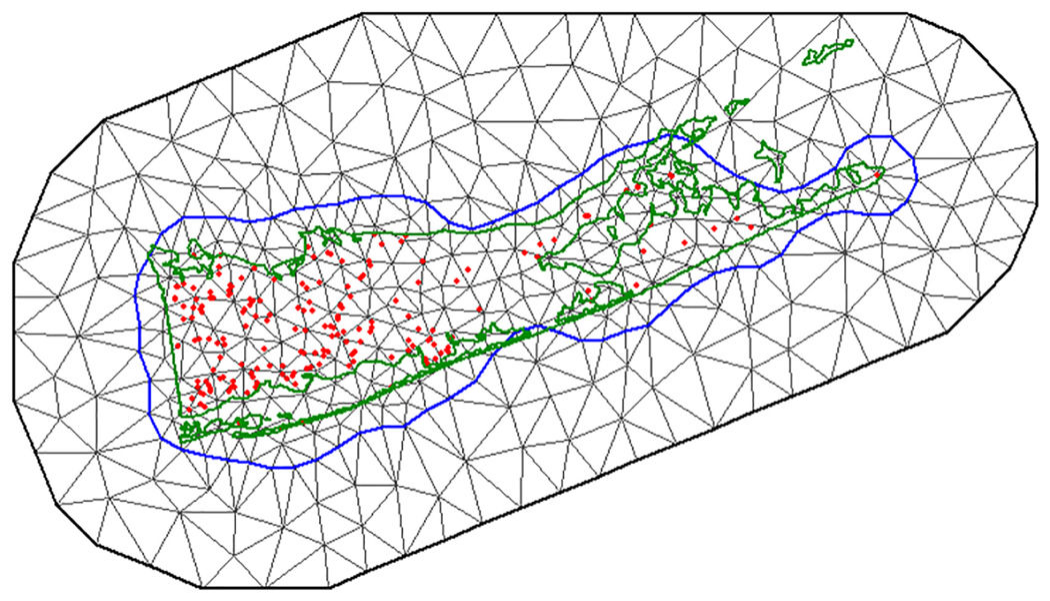
Triangulation mesh used to obtain the spatial covariance, with the political boundary of Suffolk County in green and observation points in red. The blue line represents the mesh boundary between the inner mesh, which contains the area to be modeled, and the outer mesh that is added on by the INLA mesh creator function to avoid boundary effects.

We assessed time lags of meteorological variables and use of spatial and temporal effects using the INLA SPDE (Data S2), and ranked models using a modification of the information-theoretic model selection approach (Burnham et al. 2011). Rather than the more common Akaike’s information criterion, we used the analogous deviance information criterion (DIC), which is a generalized form for hierarchical modeling (Zhu and Carlin 2000). Models with a lower DIC were considered a better fit. Spatial covariance was addressed using the SPDE model, which calculates a Gaussian Markov random field based on a triangulation mesh overlaid on the study area domain (Fig. 1). In brief, the SPDE evaluates spatial covariance as an underlying continuous Gaussian surface (which is very high cost to calculate over any sizeable area), by utilizing a link function that allows substitution of a Gaussian Markov random field which is discretely indexed and far less computationally complex (Lindgren et al. 2011, Blangiardo and Cameletti 2013). The triangular mesh is then used to construct an observation matrix, referred to as A in the R-INLA package, that contains the values of the spatial random field across the study area. An in-depth description of the relevant equations and hierarchical model structure can be found in Musenge et al. (2013) and Cosandey-Godin et al. (2014). For this study, the default non-informative prior distributions were used for all regression coefficients and hyperparameters, allowing our large number of observations (*n* = 10,686) to inform the posterior distributions. Briefly, in Bayesian modeling, prior distributions are assigned to reflect the researcher’s a priori knowledge of the values and variability that a model parameter might take. The non-informative priors we used assume very little a priori knowledge, allowing the properties of the data to predominate in calculating the posterior distributions by assigning equal prior probability to all outcomes. We used the INLA default priors, which in the case of the SPDE take the form of Gaussian distributions whose mean and variance are calculated based on the size of the study area (Cameletti et al. 2013), and in the case of the regression coefficients are Gaussian priors with mean zero and fixed variance 10,000, that is, a flat prior. The R code used to set the SPDE priors can be found in Data S2. Temporal covariance was addressed using a first-order temporal autoregressive process (AR1), which models WNV presence at a trap site as a function of presence in the previous week plus an error term (Potzelberger 1990). The precision and autoregressive parameter (φ) for week is reported for each model run.

Our response, WNV presence/absence, was coded as a binary outcome. Logistic regression is typically used to predict binary responses and produces a logit-linear measure of the probability of a positive outcome. We elected to use a beta-binomial-likelihood model to account for overdispersion caused by the large number of zeroes in our response variable. Details of the beta-binomial function used by R-INLA can be found in the online project documentation (R-INLA Project 2016). The overdispersion parameter was reported for each model run, along with its 95% credible interval. If the 95% credible interval for the overdispersion parameter does not include zero, there is evidence of overdispersion.

Predictive power was tested by holding out the data for the most recent year available (2015) during all steps of variable selection and calibration of the model. We then computed predicted values for the holdout year and performed a sensitivity analysis using a range of decision values to determine the binary presence/absence of WNV from the probabilities provided by model predictions and determined the optimal decision rule for classifying a predicted observation. We chose to test the model on the most recent available year’s data rather than a holdout subset from the entire dataset in order to simulate practical application of the model, predicting future hotspots of WNV mosquito infection in a year for which the model is naive. Sensitivity analysis was conducted by producing a receiver-operating characteristic (ROC) curve using the R package “ROCR,” (Sing et al. 2005) which plots the sensitivity (true-positive rate) against 1-specificity (false-positive rate), across all possible cutoff points. The area under curve (AUC) value was computed by integrating the ROC curve, and serves as a diagnostic of overall predictive accuracy. The AUC value can be summarized as the probability that a randomly chosen WNV-positive observation will have a higher modeled value than a randomly chosen WNV-negative observation. An AUC value that deviates from 0.5 indicates that the model is better than random chance at predicting the outcome. The optimized cutoff point to minimize false positives and maximize true positives was determined as the point along the ROC curve with maximal value of the Youden’s index (Fluss et al. 2005). Youden’s index, or the *J* statistic, is calculated as *J* = sensitivity + specificity − 1, and measures the performance of a dichotomous classifier.

Analysis was performed using R version 3.2.3 statistical software (R Development Core Team 2016) on a Dell Latitude E7240 laptop computer with an Intel Core i5–4300U CPU and 8 GB of RAM, running Windows 7 Enterprise. R code for preparing the models and creating the figures in this paper is available in Data S2. Supplemental data for running the R code are available in Data S3, and the shapefile for constructing the INLA triangulation mesh is available in Data S4.

## Results

The correlation matrix identified 12 groups of correlated variables (Data S1, listed below correlation matrix). One variable from each group was selected for inclusion in the PCA based on strength of correlation to the result, relevance to the research question, and professional judgment. Eight variables were not correlated with any other to a significant degree and were included as well, for a total of 20 variables included in the PCA.

**Fig. 2.**
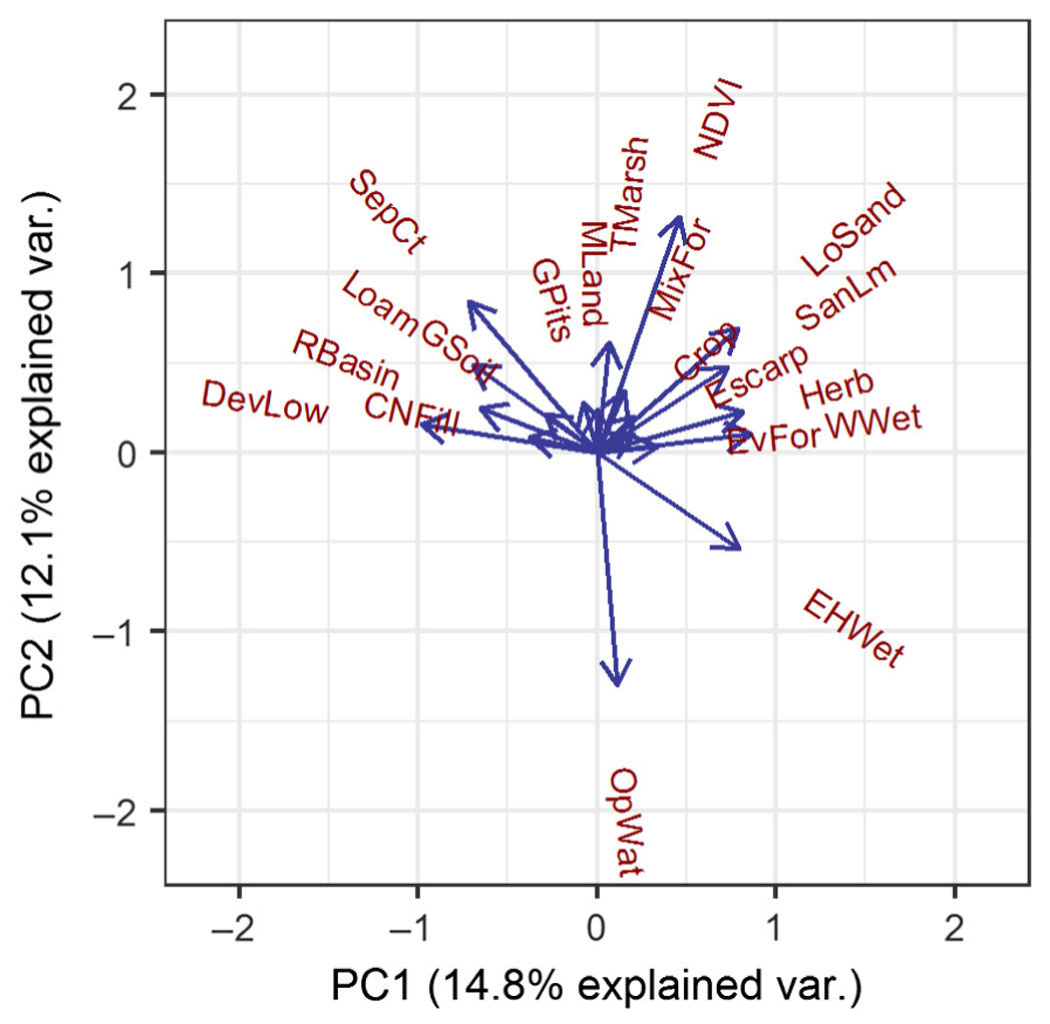
Principal component analysis plot illustrating the relative magnitude and direction of variable loadings for the first two principal components on a subset of predictors identified during variable selection.

Principal component analysis was conducted (Fig. 2), and variable selections were made from among the first two principal components (explaining 14.8% and 12.1% of variance, respectively). Six predictors with the highest loadings in the first two principal components were selected for inclusion in the final model, for parsimony and ease in collecting data for future model application. Also included were weekly mean precipitation and temperature at 0-, one-, and two-week lag times (Table 3).

Meteorological variables were evaluated with no time lag, and at one- or two-week lag times, along with a model that included all three (Table 4). Comparing the models by DIC, the best model included precipitation and temperature variables at no lag time. However, we chose to include the model that incorporated all three meteorological lag times, because including lagged meteorological variables dramatically reduced the error attributed to spatial effects, reducing both the spatial variance and correlation range. Average temperature had a strong positive relationship with WNV (>95% credible interval [CI] above zero) at two-week lag times, with a decreasing, but still positive strength of effect at lesser lag times. Precipitation with no time lag exhibited a positive relationship with WNV, while precipitation at one-week lag time showed a strong negative relationship (>95% CI below zero). At two-week lag time, a weak negative relationship with WNV was observed. Comparing overdispersion and the AR(1) parameter among lag times revealed no differences.

**Fig. 3.**
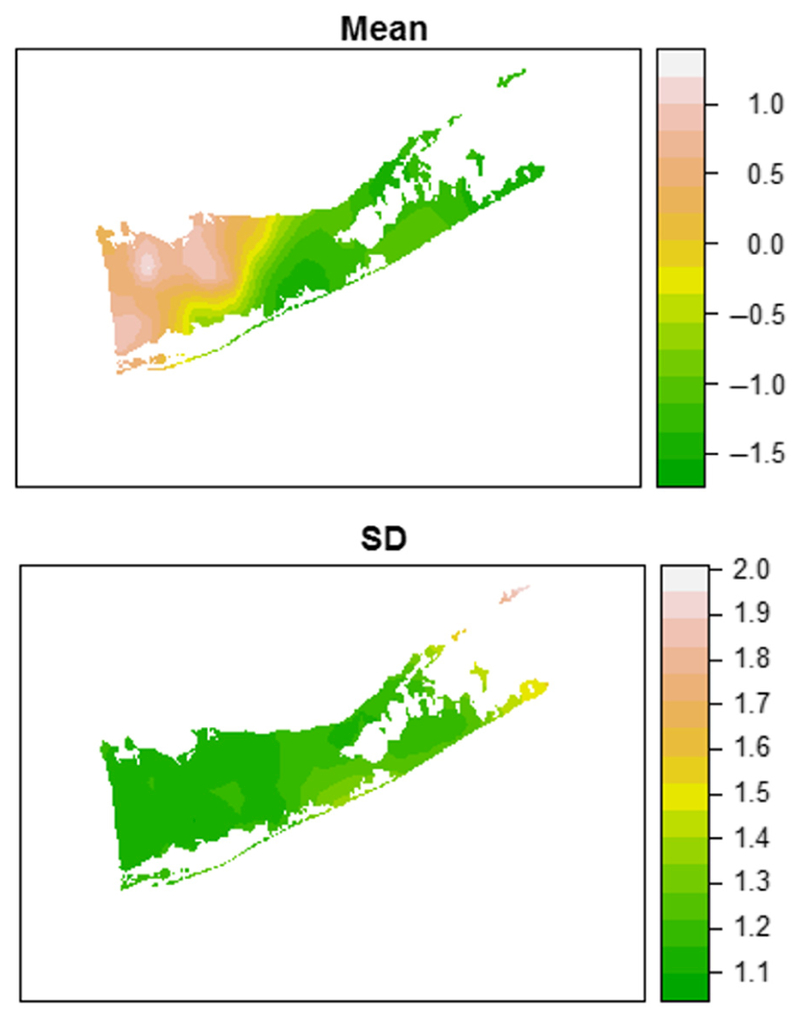
Mean and standard deviation of stochastic partial differential equation spatial effect on West Nile virus (WNV) infection in Suffolk County, New York, USA. Units are presented as log-odds. The odds of WNV mosquito infection grow higher in a gradient from east to west on the island. Variability is largely homogenous throughout the study area, with the exception of points on the extreme ends of the North and South Forks that had few observations.

Spatial and temporal random effects were evaluated individually and jointly for inclusion in the model (Table 5). Based on DIC comparison, adding the spatial effect resulted in a large improvement to the model fit (ΔDIC = 7325.58). The model with an AR(1) effect was a further improvement relative to the spatial model (ΔDIC = −216.12), and the spatiotemporal model with both effects included was the best-fit model evaluated (ΔDIC relative to the temporal model = −187.62). Posterior distributions for the covariates differed between the models, indicating that the errors have a spatial and temporal structure that was being ignored in the non-spatiotemporal model. In the full spatiotemporal model, the posterior distribution of the AR(1) parameter indicated a strong autocorrelation effect (95% CI = 0.94:0.99). The variance of the spatial effect showed a wide posterior distribution (95% CI = 0.38:3.14). While this value cannot be directly compared to the linear predictors, we note that a significant spatial effect is present in the data. The posterior mean of the range parameter, indicative of the distance at which the mean spatial variance declines to approximately 0.13, was 79 km (95% CI 31:183). The posterior mean and SD of the spatial effect were plotted across the study area (Fig. 3). The spatial effect exhibited an increasing trend from the eastern to western side of the county and was most pronounced in the northwestern area near the Town of Huntington. Standard deviations were high (mean SD 1.46) compared to the mean values of the spatial effect, indicating a high degree of variation was present. The SD was consistent across the majority of the study area, with small pockets of higher variation at the extreme eastern ends of the North and South Forks.

Of the final set of variables chosen for inclusion in the model, SepCt, OpWat, WWet, TAvgL2, Prcp, and PrcpL1 were significantly associated with WNV presence at the 5% level. Posterior distributions for these variables are presented in Table 5 as log-odds of scaled variables, meaning that interpretation of each coefficient is dependent on the magnitude and distribution of each variable (Table 3). The odds ratios presented here represent the degree of change in odds resulting from a change of one SD from the mean in the variable under consideration. The percentage of NLCD (National Land Cover Dataset) pixels classified as open water within a 1-km circular buffer, or OpWat, had the highest magnitude of effect (odds ratio 0.41, 95% CI 0.29:0.58), followed by the mean weekly temperature at two-week lag time, TAvgL2 (OR 1.34, 95% CI 1.16:1.55) and the count of septic systems within a 1-km circular buffer, SepCt (OR 1.28, 95% CI 1.11:1.51). The presence of woody wetlands and the weekly mean precipitation at one-week lag time had a negative association with WNV presence to a smaller, but still significant degree. Precipitation at no lag time had a small, significant positive association with WNV infection. Despite the 95% CI posterior distributions for NDVI, DevLow, EHWet, PrcpL2, Tavg, and TavgL1 encompassing zero (analogous to odds ratio CI that encompass one), they were left in the model as post hoc manipulation of the predictors would bias the model selection process.

**Fig. 4.**
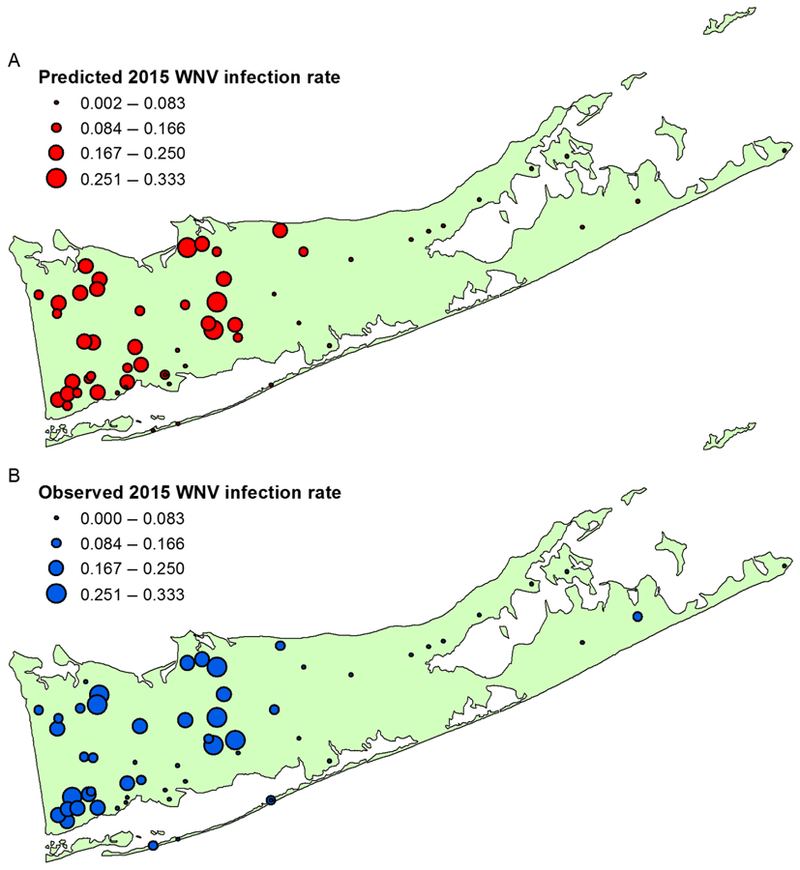
Predicted (A) and observed (B) West Nile virus (WNV) infection rates from traps in the 2015 holdout dataset. Rates listed reflect the mean annual frequency of a mosquito pool testing positive for WNV.

**Fig. 5.**
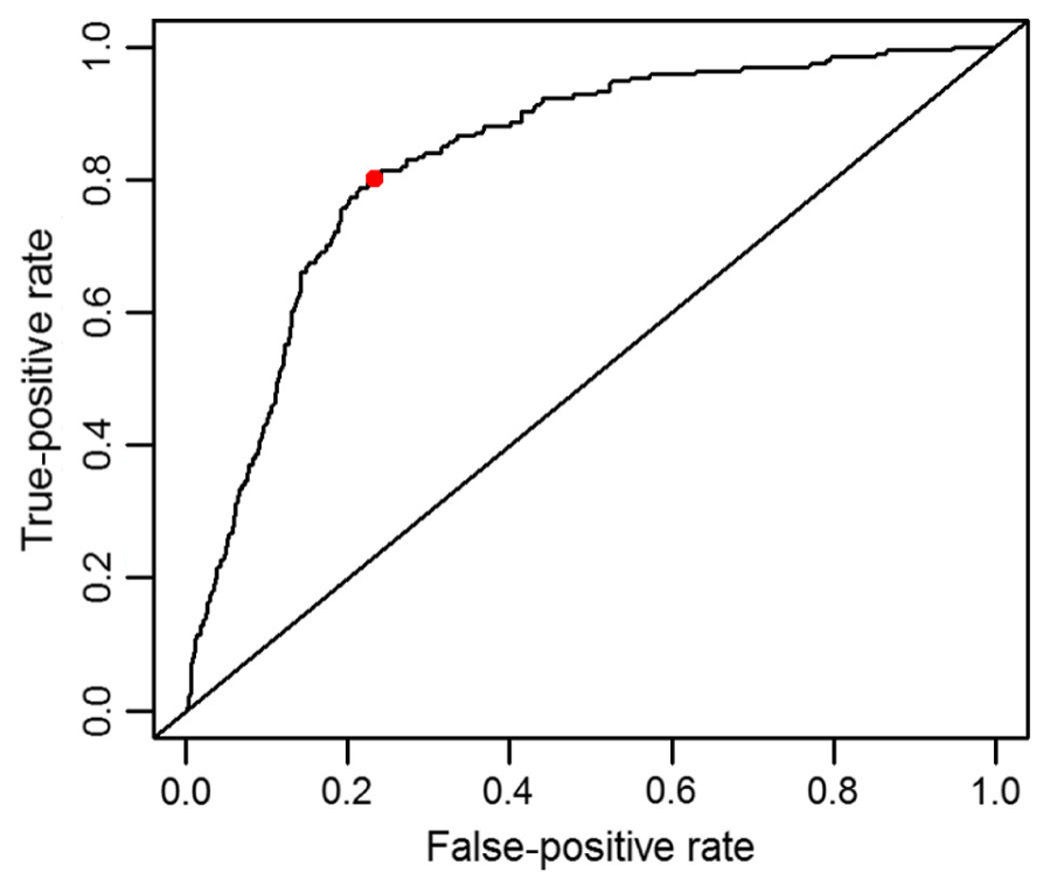
Receiver-operating characteristic curve for the final spatiotemporal model predicting 2015 West Nile virus incidence, illustrating the tradeoff between sensitivity and specificity at a variety of cutoff points. The optimal cutoff, illustrated with a dot, minimizes the distance between the curve and the upper left corner of the graph, which represents a 100% accuracy rate.

In order to test the predictive power of the full, final spatiotemporal model, predictions were made for the holdout dataset in 2015 with *n* = 1366 observations (Fig. 4). The ROC curve (Fig. 5) indicated that the model has predictive power well above random chance, depicted by the diagonal line. The AUC value was found to be 0.834, confirming that the model is better than random chance at classifying WNV-positive mosquito pools. The optimal cutoff point, maximizing Youden’s index, was 0.178, resulting in a sensitivity of 0.809 and a specificity of 0.770.

## Discussion

This study examined the spatiotemporal associations of WNV infection in *Culex pipiens–restuans* mosquitoes, along with the effects of several land-cover, meteorological, and sociological variables in Suffolk County, New York. Variables were chosen from many easily obtainable public data sources and were selected by a multi-step process. We identified several variables that had an effect on the prediction of West Nile infection, along with a gradient of spatial effect increasing from the eastern to western side of the county and a strong degree of temporal autocorrelation. The resulting model was able to predict West Nile presence in a holdout year with a reasonable degree of accuracy while avoiding excessive false positives. Our variable and spatial results con-firmed the results of previous studies in the region, and uncovered a positive association between septic systems and WNV that was previously seen only in the tropics.

The posterior distributions of covariate effect sizes show that, of variables tested, the percent NLCD coverage of open water within a 1-km buffer has the largest effect on *C. pipiens–restuans* WNV incidence in Suffolk County. The strong negative association reflects the life history of the *C. pipiens–restuans* complex as container-breeding freshwater mosquitoes (Vinogradova 2000). Large bodies of water, either salt or fresh, favor the production of floodwater mosquitoes, which are a nuisance but not significant vectors of WNV in Suffolk County. *Culex pipiens–restuans* mosquitoes preferentially lay their eggs in temporary bodies of water in order to avoid predators that are more often present in permanent water bodies (Blaustein et al. 2005, Walton et al. 2009). The presence of temporary water may also contribute to WNV spillover from infected mosquitoes. A serological survey study conducted in southern France found that the risk of WNV seropositivity in horses was elevated in areas that experienced variations in open water and wetland coverage between winter and summer (Pradier et al. 2014). The surveyors proposed that the presence of temporary water in late winter and early spring (March–July) fosters the endemic transmission of WNV to and from birds and mosquitoes, and decreasing open water areas in summer lead to a congregation of susceptible birds at high density at the time when mosquito populations are at their highest. Research conducted in Florida examining the connection between drought and epizootic cycling of WNV in chickens further supports the hypothesis that the concentration of avian hosts and active mosquitoes around water sources at the height of the mosquito breeding season fosters greater amplification of the disease (Shaman et al. 2005).

Mean temperature, especially at two-week lag time, had a strong positive association with WNV prevalence, while the lagged mean precipitation had a smaller negative association. These results echo earlier findings in Suffolk County that found that lower precipitation coupled with high temperatures increases WNV infection in mosquitoes in the northeastern United States (Shaman et al. 2011, Little et al. 2016). High temperature favors the survival of *Culex* mosquito larvae and shortens the time they spend in the larval and pupal stages, while increasing biting rates and hence the rate of infection (Ruiz et al. 2010). High precipitation can have the effect of flushing mosquito larvae and pupae out of containers and lowering the overall number that survive to become adults, and *C. pipiens* are particularly vulnerable (Koenraadt and Harrington 2008). Precipitation in the same week a mosquito pool was collected, however, was weakly positive in association with WNV infection. This may reflect that in the short term, temporary increases in the water volume of containers can increase larval development rates and decrease overall mortality by lowering population density (Olejnicek and Gelbic 2000). It may be that the effect of precipitation on WNV infection rates is nonlinear, with small amounts of precipitation encouraging mosquito survival and WNV transmission and larger downpours leading to a flushing effect. The effect of precipitation on mosquito population is complex and the optimal lag time for modeling can vary over time even in the same study area (Ruiz et al. 2010). It is therefore important to reevaluate meteorological variables each time a mosquito WNV infection model is calibrated. The presence of woody wetlands, and to a smaller degree, emergent herbaceous wetlands, had a negative association with WNV infection, in agreement with earlier work that found a similar association in the northeastern United States (Bowden et al. 2011). Wetland areas can provide the ecosystem service of controlling WNV infection risk by naturally attenuating mosquito populations by harboring predators (Walton et al. 2009) and preserving healthy avian community composition, which reduces the density of competent viral hosts (Ezenwa et al. 2007). We further propose that non-seasonal wetlands can function much like permanent open water areas in reducing the number of endemic transmission events between birds and mosquitoes by providing greater habitat area and reducing overall population density.

The number of septic systems in a 1-km radius was a variable of interest prior to model specification, due to the unusual density of older septic and cesspool systems in Suffolk County and the presence of sewered areas in proximity to entirely unsewered areas allowing comparison between the two. We found that higher septic system density was a strong predictor of WNV infection, echoing results from studies conducted in Puerto Rico (Barrera et al. 2008, Burke et al. 2010) that found that septic tanks can provide a breeding area and larval shelter, producing large numbers of mosquitoes. In a subsequent study, an association was found between times of peak mosquito production in human-managed containers including septic systems and peak dengue fever incidence (Barrera et al. 2011). By providing a sheltered habitat free of predators, septic systems that become unsealed either through structural failure or through a lack of protective mesh on inspection ports are conducive to increased mosquito population density, increasing the probability of WNV transmission from avian hosts.

Potential sources of error in the present study include spatial imprecision in rasterized sources of remote-sensed geospatial data, cloud coverage in remote-sensed data, and loss of resolution in time-averaged meteorological models. Land-cover data and NDVI measurements were derived from 30-m rasterized datasets with a circular 1-km buffer applied around each trap site. The application of a circular buffer to raster data introduces small errors at the buffer boundary by blurring the edges of the circle to accommodate the square raster pixels. For the purposes of this study, cells with >50% inclusion in the circular buffer were considered within the buffer. For NDVI measurement, scenes without cloud cover were often not available for a given area. For each year, the least cloudy measurement according to the reported LANDSAT-8 cloud cover measurements was used, but in any satellite-sensed dataset, there is potential for error from cloud cover. The Daymet dataset is modeled using measurements from NOAA (National Oceanic and Atmospheric Administration) weather stations and may include some discrepancy from the actual meteorological conditions at each trap site. Additionally, in order to fit the weekly temporal resolution of trap site sampling, Daymet data were averaged weekly, leading to a loss of resolution.

## Conclusions

We investigated WNV incidence in Suffolk County, New York, mosquitoes using predictors from publicly available quality-controlled datasets. Using the recently developed INLA SPDE statistical method, we included continuous spatial effects and temporal autocorrelation. Our study confirms previous associations found in WNV models, including the link between low precipitation, high temperatures, and WNV incidence. We found that woody wetlands are associated with reduced WNV in mosquito populations and that the density of septic systems predicts an increase in WNV. This model will allow estimation of WNV incidence in mosquitoes using a small set of easily obtained predictors, with applications in vector control/management and the prioritization of geographical areas for public health intervention.

We recommend this methodology for datasets involving the prediction of rare events over large study areas, where the spatial relationship to the result is of particular interest. Rare events modeling (such as disease ecology or prediction of natural disasters) suffers from clustering or binning of responses, which sacrifices precision. The INLA SPDE method allows users to create a map of the spatial covariate over an arbitrarily fine-grained raster due to the use of a continuous Gaussian random field. This approach is a clear improvement over spatial models that must grid or cluster responses. Additionally, INLA is a computationally inexpensive method of incorporating both spatial and temporal effects into mixed models. Spatiotemporal model fitting is typically complex to implement and requires a powerful computer, a long run time, or both. INLA runs entirely within the commonly used R statistical software and is relatively simple to implement with intermediate levels of programming expertise. The approach shows promise for developing surveillance and control programs. We encourage future research in disease ecology and rare events prediction to consider the INLA SPDE approach for spatiotemporal mixed modeling.

## Supplementary Material

1

2

3

4

## Figures and Tables

**Table 1.** West Nile virus (WNV) surveillance in *Culex* mosquitoes, Suffolk County, New York, USA, 2008–2015.

Years	No. of pools tested	No. of individuals tested	WNV-positive pools
2008	1164	38,503	41
2009	1276	46,143	17
2010	1678	47,291	289
2011	1553	60,391	79
2012	1075	43,989	206
2013	1300	53,653	176
2014	1347	56,743	186
2015	1350	50,981	197

**Table 2.** Variables considered for inclusion.

Sources	Variables	Codes	Description
Daymet	Weekly mean precipitation	Prcp	Weekly mean precipitation at trap site (mm/d)
	Weekly mean temperature	Tavg	Weekly mean two-meter air temperature (°C)
	Lag 1 weekly mean precipitation	PrcpL1	“ ”, lagged 1 week
	Lag 1 weekly mean temperature	TavgL1	“ ”, lagged 1 week
	Lag 2 weekly mean precipitation	PrcpL2	“ ”, lagged 2 weeks
	Lag 2 weekly mean temperature	TavgL2	“ ”, lagged 2 weeks
Suffolk County	Septic count	SepCt	Estimated number of septic systems within 1 km (count)
Landsat	Normalized Difference Vegetation Index	NDVI	Mean index of summer vegetation cover within 1 km (0–1)
NLCD	Open water	OpWat	Percentage of pixels classified open water within 1 km (0–1)
	Developed, Open space	DevOpen	“ ”
	Developed, Low intensity	DevLow	“ ”
	Developed, Medium intensity	DevMed	“ ”
	Developed, High intensity	DevHi	“ ”
	Barren land	Barr	“ ”
	Deciduous forest	DecFor	“ ”
	Evergreen forest	EvFor	“ ”
	Mixed forest	MixFor	“ ”
	Shrub and scrub	ShrScr	“ ”
	Herbaceous	Herb	“ ”
	Hay pasture	Hay	“ ”
	Cultivated crops	Crop	“ ”
	Woody wetlands	WWet	“ ”
	Emergent herbaceous wetlands	EHWet	“ ”
SSURGO	Beach	Bch	Percentage of pixels classified Beach within 1 km (0–1)
	Tidal Marsh	TMarsh	“ ”
	Water	Wat	“ ”
	Dunes	Dune	“ ”
	Muck	Muck	“ ”
	GravelPits	GPits	“ ”
	Graded soil	GSoil	“ ”
	Made land	MLand	“ ”
	Mucky sand	MSand	“ ”
	Dredged material	Dredge	“ ”
	Urban land	UrbLand	“ ”
	Recharge basin	RBasin	“ ”
	Dune land	DLand	“ ”
	Escarpments	Escarp	“ ”
	Sandy loam	SanLm	“ ”
	Silty loam	SilLm	“ ”
	Haven soil	HSoil	“ ”
	Sand	Sand	“ ”
	Loamy sand	LoSand	“ ”
	Loam	Loam	“ ”
	Cut and fill	CNFill	“ ”

*Note:* NDVI, Normalized Difference Vegetation Index; SSURGO, U.S. Department of Agriculture Soil Survey Geographic.

**Table 3.** Summary statistics for variables considered for final model.

Variables	Units	Mean (SD)	Range
OpWat	% pixels in 1-km buffer	0.10 (0.20)	0.00:0.73
EHWet	% pixels in 1-km buffer	0.03 (0.05)	0.00:0.30
WWet	% pixels in 1-km buffer	0.03(0.06)	0.00:0.47
DevLow	% pixels in 1-km buffer	0.25 (0.16)	0.00:0.73
SepCt	No. of tanks in 1-km buffer	702.1 (692.2)	0.0:2581.0
NDVI	Unitless index (0–1)	0.58 (0.10)	0.33:0.78
^TAv^g	Degrees Celsius	22.09 (2.81)	8.03:28.96
TAvgL1	Degrees Celsius	22.21 (2.65)	13.25:28.96
TAvgL2	Degrees Celsius	22.08 (2.68)	13.32:28.96
Prcp	Millimeters rainfall	3.2 (4.0)	0.0:33.0
PrcpL1	Millimeters rainfall	3.2 (4.0)	0.0:33.0
PrcpL2	Millimeters rainfall	3.4 (4.2)	0.0:33.0

*Note*: NDVI, Normalized Difference Vegetation Index; SD, standard deviation.

**Table 4.** Spatiotemporal regression results of time-lagged models.

Variables	No lag	One-week lag	Two-week lag	All lags included
SepCt	0.22 (0.08:0.37)	0.23 (0.09:0.40)	0.25 (0.10:0.41)	0.25 (0.10:0.41)
NDVI	−0.12 (−0.32:0.08)	−0.14 (−0.34:0.07)	−0.13 (−0.33:0.08)	−0.12 (−0.32:0.09)
OpWat	−0.91 (−1.27:−0.56)	−0.92 (−1.29:−0.57)	−0.90 (−1.27:−0.55)	−0.88 (−1.24:−0.54)
DevLow	−0.07 (−0.27:0.12)	−0.08 (−0.29:0.11)	−0.09 (−0.30:0.11)	−0.08 (−0.29:0.11)
WWet	−0.15 (−0.30:−0.01)	−0.14 (−0.29:−0.01)	−0.14 (−0.29:−0.01)	−0.14 (−0.30:0.00)
EHWet	−0.01 (−0.18:0.17)	−0.02 (−0.19:0.16)	−0.02 (−0.19:0.16)	−0.02 (−0.19:0.15)
Prcp	0.10 (0.02:0.17)			0.10 (0.02:0.17)
PrcpL1		−0.11 (−0.20:−0.02)		−0.11 (−0.20:−0.03)
PrcpL2			−0.04 (−0.13:0.04)	−0.06 (−0.14:0.02)
^Tav^g	0.19 (0.05:0.34)			0.08 (−0.07:0.23)
TavgL1		0.29 (0.15:0.42)		0.14 (−0.01:0.28)
TavgL2			0.37 (0.24:0.51)	0.29 (0.15:0.44)
Parameter				
Overdispersion	0.50 (0.04:0.96)	0.50 (0.04:0.96)	0.50 (0.04:0.96)	0.50 (0.04:0.96)
$\sigma_{\text{s}}^{2}$	5.78 (1.61:15.39)	1.29 (0.40:3.36)	1.26 (0.39:3.28)	1.22 (0.38:3.14)
* r*	194 (98:354)	82 (32:191)	80 (31:185)	79 (31:183)
φ (AR1)	0.92 (0.80:0.98)	0.98 (0.94:0.99)	0.98 (0.93:0.99)	0.98 (0.94:0.99)
Effective no. parameters	41.35 (3.526)	42.70 (4.07)	43.10 (4.06)	46.28 (4.12)
DIC	4556.42	4631.76	4625.16	4615.77

*Notes*: Error is expressed as either 95% credible interval or SD. AR1, autoregressive process; DIC, deviance information criterion; NDVI, Normalized Difference Vegetation Index; SD, standard deviation.

**Table 5.** Regression results of models employing different error structures.

Variables	No spatial or temporal random errors	Spatial model	Temporal model	Spatiotemporal model
SepCt	0.18 (0.12:0.23)	0.24 (0.11:0.38)	0.27 (0.17:0.36)	0.25 (0.10:0.41)
NDVI	−0.19 −0.25:−0.13)	−0.05 (−0.24:0.14)	−0.31 (−0.41:−0.20)	−0.12 (−0.32:0.09)
OpWat	−0.23 (−0.29:−0.18)	−0.84 (−1.17:−0.52)	−1.33 (−1.59:−1.09)	−0.88 (−1.24:−0.54)
DevLow	−0.02 (−0.07:0.03)	−0.07 (−0.25:0.10)	0.25 (0.13:0.36)	−0.08 (−0.29:0.11)
WWet	0.01 (−0.05:0.05)	−0.20 (−0.34:−0.06)	0.05 (−0.05:0.14)	−0.14 (−0.30:0.00)
EHWet	−0.07 (−0.12:−0.02)	−0.03 (−0.19:0.13)	−0.39 (−0.54:−0.24)	−0.02 (−0.19:0.15)
Prcp	0.03 (−0.01:0.07)	0.14 (0.08:0.21)	0.11 (0.04:0.18)	0.10 (0.02:0.17)
PrcpL1	−0.09 (−0.13:−0.04)	−0.10 (−0.19:−0.02)	−0.12 (−0.21:−0.04)	−0.11 (−0.20:−0.03)
PrcpL2	−0.05 (−0.09:−0.01)	0.06 (−0.02:0.14)	−0.05 (−0.13:0.03)	−0.06 (−0.14:0.02)
^Tav^g	−0.01 (−0.07:0.04)	−0.14 (−0.25:−0.04)	0.11 (−0.04:0.25)	0.08 (−0.07:0.23)
TavgL1	0.10 (0.03:0.17)	0.25 (0.13:0.37)	0.17 (0.02:0.32)	0.14 (−0.01:0.28)
TavgL2	0.24 (0.19:0.30)	0.78 (0.68:0.89)	0.36 (0.21:0.50)	0.29 (0.15:0.44)
Parameter				
Overdispersion	0.50 (0.06:0.94)	0.50 (0.04:0.96)	0.50 (0.04:0.96)	0.50 (0.04:0.96)
$\sigma_{\text{s}}^{2}$		3.49 (1.25:8.50)		1.22 (0.38:3.14)
*r*		191 (112:326)		79 (31:183)
φ (AR1)			0.98 (0.94:0.99)	0.98 (0.94:0.99)
Effective no. parameters	12 (0.00)	25.31 (3.13)	25.56 (1.01)	46.28 (4.12)
DIC	12345.09	5019.51	4803.39	4615.77

*Notes*: Error is expressed as either 95% credible interval or SD. AR1, autoregressive process; DIC, deviance information criterion; NDVI, Normalized Difference Vegetation Index; SD, standard deviation.
