# Supplementary material for: Spatiotemporal modeling of ecological and sociological predictors of West Nile virus in Suffolk County, NY, mosquitoes
Source: Ecosphere. Author manuscript; Available in PMC 2018 Aug 22. (PMC6104833; doi:10.1002/ecs2.1854)
Supplement: 1 [file NIHMS983434-supplement-1.zip › Myer ECS17-0061R1 Metadata S1.pdf]

# Spatiotemporal modeling of ecological and sociological predictors of West Nile virus in Suffolk County, NY mosquitoes

Mark H. Myer[1], Scott R. Campbell[2], John M. Johnston[1]†

[1]US Environmental Protection Agency, Office of Research and Development, National Exposure Research Laboratory. 960 College Station Rd, Athens, GA, United States 30605.
[2]Arthropod-Borne Disease Laboratory, Suffolk County Department of Health Services. Yaphank, NY, United States 11980-9744.

**† E-mail:** Johnston.JohnM@epa.gov

**Data S1: Correlation matrix for variable selection.**
Author: Mark H. Myer
Files included: DataS1CorrMatrix.csv
Description: This file contains a correlation matrix that was used to identify and select correlated variables in the development of the finalized INLA model. The groups of correlated variables are identified below the main body of the matrix, and the variable selected from among each group is identified.
