# Supplementary material for: Spatiotemporal modeling of ecological and sociological predictors of West Nile virus in Suffolk County, NY, mosquitoes
Source: Ecosphere. Author manuscript; Available in PMC 2018 Aug 22. (PMC6104833; doi:10.1002/ecs2.1854)
Supplement: 2 [file NIHMS983434-supplement-2.zip › Myer ECS17-0061R1 Metadata S2.pdf]

patiotemporal modeling of ecological and sociological predictors of West Nile virus in Suffolk County, NY mosquitoes

Mark H. Myer[1], Scott R. Campbell[2], John M. Johnston[1]†

[1]US Environmental Protection Agency, Office of Research and Development, National Exposure Research Laboratory. 960 College Station Rd, Athens, GA, United States 30605.
[2]Arthropod-Borne Disease Laboratory, Suffolk County Department of Health Services. Yaphank, NY, United States 11980-9744.

† E-mail: Johnston.JohnM@epa.gov

Data S2: R code for running INLA-SPDE model and reproducing figures
Author: Mark H. Myer
Files included: DataS2_INLAModel.R
Description: Commented code that details how to use the included dataset to reproduce the statistical analysis and figures in the manuscript. An interested reader should use this code along with Data S3 in order to reproduce this study's findings and figures.
