# Supplementary material for: Spatiotemporal modeling of ecological and sociological predictors of West Nile virus in Suffolk County, NY, mosquitoes
Source: Ecosphere. Author manuscript; Available in PMC 2018 Aug 22. (PMC6104833; doi:10.1002/ecs2.1854)
Supplement: 3 [file NIHMS983434-supplement-3.zip › Myer ECS17-0061R1 Metadata S3.pdf]

# Spatiotemporal modeling of ecological and sociological predictors of West Nile virus in Suffolk County, NY mosquitoes

Mark H. Myer[1], Scott R. Campbell[2], John M. Johnston[1]†

[1]US Environmental Protection Agency, Office of Research and Development, National Exposure Research Laboratory. 960 College Station Rd, Athens, GA, United States 30605.
[2]Arthropod-Borne Disease Laboratory, Suffolk County Department of Health Services. Yaphank, NY, United States 11980-9744.

**† E-mail:** Johnston.JohnM@epa.gov

**Data S3: Dataset including mosquito trap WNV testing results and variables**
Authors: Mark H. Myer, Scott R. Campbell
Files included: DataS3_Dataset.csv
Description: This dataset includes all data needed to reproduce the statistical analysis and figures in this study, including WNV testing results, trap location coordinates, and all variables considered. An interested reader should use this data along with the code in Data S2 to reproduce this study.
